# New trends for anemia at the beginning of a new century…

**DOI:** 10.1590/S1516-31802004000600001

**Published:** 2004-11-04

**Authors:** Isabela M. Benseñor

In a recent article published in the Archives of Internal Medicine,^1^ the authors comment that "for too long, anemia has been viewed as an innocent bystander" in its frequent association with chronic diseases. How can we interpret anemia in Brazil, at this moment?

First, long-term time trends in Brazil, based on three cross-sectional studies conducted in the 1970s, 80s, and 90s, show us a rapid decline in the prevalence of childhood malnutrition and a rapid increase in adult overweight/obesity, but a high prevalence of anemia with a possible epidemic trend among children aged under five.^2^ Recent data suggest that the prevalence of anemia is increasing in all regions in the country, including richer and poorer areas, with the same burden. One possible cause for this, among children aged under two, is thought to be the large increase in milk consumption.^3^ Recent data on the prevalence of anemia among adults are more sparse: there are just two studies, from Pernambuco and Piauí, which simultaneously evaluated children and adults and found a nearly 25% prevalence of anemia among adults.^4^

Second, the diagnosis of anemia of moderate severity (9-13 g/dl) is very complicated because of our low perception of mild pallor at physical examination. Several data from the literature confirm this point.^5-7^ In Brazil, the reliability and validity of the presence of palmar and conjunctival pallor for anemia detection purposes have been evaluated by Spinelli et al.^8^ The results from comparing anemia diagnoses made by palmar and conjunctival examination with hemoglobin levels determined by blood cell count showed low levels of agreement. Conjunctival pallor was more sensitive than palmar pallor for diagnosing anemia. The authors concluded that it was still too early to recommend routine use of this technique without any kind of confirmation. Because of this difficulty in evaluating anemia in a clinical examination, several papers have discussed simple methods for diagnosing anemia in African countries where blood count tests are an impossibility. New strategies have been proposed, including hemoglobin color scales on which a drop of blood is compared by means of a plastic scale of red circles with the hemoglobin levels that these represent.^6,9^

Recent data have shown that quantifying anemia by choosing between four levels is a very imprecise method with no interobserver reliability. It is more correct to classify the grade of pallor as slight, moderate or severe or, better, as presence or absence of pallor.^10^ Such difficulty will be probably more intense for hemoglobin levels of 9-13 g/dl.

Recent data from Hospital das Clínicas, Faculdade de Medicina da Universidade de São Paulo, have shown that, out of 95 patients evaluated at an outpatient clinic, four diagnoses of anemia were made by physical examination. Of these 95 patients, and in addition to the four patients with anemia, another 31 patients were submitted to a complete blood count for various reasons other than anemia. Of the 35 complete blood counts, 11 showed anemia (the four diagnosed by physical examination and another seven for whom no pallor was observed during physical examination): one case of megaloblastic anemia, five cases of iron deficiency anemia and five cases of anemia associated with chronic disorders. All these patients had hemoglobin levels between 9 and 13 g/dl (Benseñor et al.; unpublished data).

The treatment of megaloblastic and iron deficiency anemia is very simple. However, for megaloblastic anemia, it needs to be determined whether the anemia is caused by a lack of folate or vitamin B_12_. For iron deficiency anemia, it needs to be determined whether blood loss is present, and one possible cause of this is colon cancer, which is frequently manifested by iron deficiency anemia.

And what about anemia that is associated with chronic disorders? The diagnoses are made but treatment is lacking. What is the problem here?

The problem is that there is now a lot of evidence suggesting that we cannot merely consider anemia to be an innocent bystander. Recent data has shown that anemia can interfere in the prognosis for chronic diseases such as heart failure and cancer, in the morbidity caused by these and other diseases and in the quality of life associated with these disorders. Mozaffarian et al.^11^ showed in a prospective, randomized study that anemia is a predictor for mortality in cases of severe heart failure. In a retrospective study of patients undergoing hemodialysis, hemoglobin levels of less than 8 g/dl were associated with a twofold increase in the probability of death, compared with patients with hemoglobin levels of more than 10 g/dl.^12^ An observational study has noted an association between hemoglobin levels of less than 10 g/dl and increased mortality rates among patients with cardiovascular disorders, thus suggesting low tolerance of anemia among such patients.^13^

Besides the impact on mortality, anemia can also influence morbidity. The treatment of anemia can improve left ventricular hypertrophy in patients with renal disease and it has a good effect on cognitive function and quality of life.^14,15^ In patients with heart failure, anemia treatment can improve symptoms and decrease the number of hospital days.^16^ Several trials are now testing the benefits of correcting anemia in patients with various types of cancer, based on the fact that for certain types of cancer, the presence of anemia can be associated with worse local and regional control and survival rates.^17,18^

Anemia can also have an impact on quality of life, as confirmed in several studies in which a questionnaire was applied to measure quality of life before anemia treatment and some months after treatment. Most of these studies have shown an improvement in quality of life, with hemoglobin levels of 8-14 g/dl.^19,20^

Thus, when we evaluate a patient in an outpatient clinic, it is now important to be sensitive towards mild cases of anemia. Perhaps we have to be less restrictive regarding complete blood cell counts precisely because we are not good enough at looking for mild pallor. This goes against what we learned at school but is perhaps more appropriate for borderline levels of anemia in this new century, with its new perception of quality of life. The other point is that our duty is not just to diagnose anemia, but also to investigate its possible causes and then to treat them.
